# Promoting Public Engagement in Palliative and End-of-Life Care Discussions on Chinese Social Media: Model Development and Analysis

**DOI:** 10.2196/59944

**Published:** 2025-03-18

**Authors:** Yijun Wang, Han Zheng, Yuxin Zhou, Emeka Chukwusa, Jonathan Koffman, Vasa Curcin

**Affiliations:** 1 Department of Population Health Sciences Faculty of Life Sciences & Medicine King's College London London United Kingdom; 2 School of Information Management Wuhan University Wuhan China; 3 Cicely Saunders Institute of Palliative Care King's College London London United Kingdom; 4 Wolfson Palliative Care Research Centre Hull York Medical School University of Hull Hull United Kingdom

**Keywords:** palliative care, end-of-life care, health promotion, social media, China, Weibo, public engagement, elaboration likelihood model, ELM

## Abstract

**Background:**

In Chinese traditional culture, discussions surrounding death are often considered taboo, leading to a poor quality of death, and limited public awareness and knowledge about palliative and end-of-life care (PEoLC). However, the increasing prevalence of social media in health communication in China presents an opportunity to promote and educate the public about PEoLC through online discussions.

**Objective:**

This study aimed to examine the factors influencing public engagement in PEoLC discussions on a Chinese social media platform and develop practice recommendations to promote such engagement.

**Methods:**

We gathered 30,811 PEoLC-related posts on Weibo, the largest social media platform in China. Guided by the elaboration likelihood model, our study examined factors across 4 dimensions: content theme, mood, information richness, and source credibility. Content theme was examined using thematic analysis, while sentiment analysis was used to determine the mood of the posts. The impact of potential factors on post engagement was quantified using negative binomial regression.

**Results:**

Organizational accounts exhibited lower engagement compared to individual accounts (incidence rate ratio [IRR]<1; *P*<.001), suggesting an underuse of organizational accounts in advocating for PEoLC on Weibo. Posts centered on PEoLC-related entertainment (films, television shows, and books; IRR=1.37; *P*<.001) or controversial social news (IRR=1.64; *P*<.001) garnered more engagement, primarily published by individual accounts. An interaction effect was observed between content theme and post mood, with posts featuring more negative sentiment generally attracting higher public engagement, except for educational-related posts (IRR=2.68; *P*<.001).

**Conclusions:**

Overall, organizations faced challenges in capturing public attention and involving the public when promoting PEoLC on Chinese social media platforms. It is imperative to move beyond a traditional mode to incorporate cultural elements of social media, such as engaging influencers, leveraging entertainment content and social news, or using visual elements, which can serve as effective catalysts in attracting public attention. The strategies developed in this study are particularly pertinent to nonprofit organizations and academics aiming to use social media for PEoLC campaigns, fundraising efforts, or research dissemination.

## Introduction

### Background

Palliative and end-of-life care (PEoLC) provides physical, psychological, and spiritual support to patients living with life-limiting conditions and terminal diseases, improving their quality of life and assisting their families [1]. Despite its importance, global public awareness and knowledge about PEoLC is limited [2-4], especially in China. Within the context of traditional Chinese culture, discussions about death and dying have historically been considered taboo [5]. As a result, individuals nearing the end of their lives often face societal and familial moral constraints, hindering their ability to autonomously make end-of-life decisions [6]. China has the largest aging population, with a notably increasing rate [7]. However, in 2015, the quality of China’s palliative care was ranked merely 71st among 80 countries or regions worldwide [8]. The coverage rate of PEoLC service in China is only 1% [9], substantially lower than in Europe, America, and other high-income regions [10]. Studies [11,12] suggested that over 80% of Chinese patients with cancer are unaware of palliative care, although the majority support it and desire more information. Hence, there is an urgent need for public education and promotional efforts about PEoLC to address the increasing unmet demands.

Social media provides a channel for PEoLC promotion and education. Kate Granger, an English geriatrician, shared her stories at the end-of-life and described the involvement of palliative care during her patient journey on Twitter (since rebranded to X), attracting around 48,000 followers [13]. Taubert et al [14] produced and delivered video resources on YouTube and Twitter to improve communication in “do not attempt cardiopulmonary resuscitation” decisions in palliative illness in 2016. After less than 6 months, the video reached nearly 100,000 hits and the comments were extremely positive and encouraging. During the COVID-19 pandemic, people actively participated in a series of tweet chats using the Twitter hashtags #comcomcovid, #PallANZ and #PalliCovid hosted by different PEoLC organizations [15,16]. These online chats helped to support both professionals and community capacity for end-of-life care within the emerging landscape of the COVID-19 pandemic. These studies indicated that social media promotion for PEoLC could help to increase public knowledge and awareness, reduce the stigma of talking about death and dying and attract more engagement. The WHO public health model of palliative care emphasizes the importance of educating both the public and professionals to facilitate palliative care integration into health care systems [17]. Social media, with its vast reach, serves as a unique platform for disseminating PEoLC-related information to a broad audience, including young people, who are often future caregivers, patients, practitioners, and policymakers. As critical stakeholders, young social media users are also highly active in disseminating information, amplifying the reach of PEoLC education. Engaging them in this dialogue not only informs their own decision-making but also promotes greater public awareness [18]. Moreover, social media helps bridge the knowledge gap between professionals and the public, making PEoLC education more accessible and aligned with contemporary public health advocacy.

However, to date, research on PEoLC promotion has predominantly focused on English-language platforms in developed countries (eg, the United States and the United Kingdom) [19]. It is essential to extend PEoLC promotion efforts through social media to underserved regions, for example, China. Social media has demonstrated effectiveness in promoting health equity in populations at risk for disadvantage [20]. Therefore, social media may consequently help address the knowledge gap of the public about PEoLC in underserved regions.

Evidence suggests that public participation in interactions on social media will increase effective cognition [21]. It has been studied that public engagement on social media can improve the awareness of health-related information, a sense of belonging, and social connection [20]. Hence, public engagement behaviors with PEoLC-related content on social media, such as commenting, liking and forwarding PEoLC posts, are likely to increase public awareness and knowledge of PEoLC and make changes in PEoLC-related behaviors. It is necessary to understand how to promote public engagement with PEoLC discussions on social media. This study aims to investigate for the first time the factors influencing public engagement of PEoLC posts and explores best practice recommendations for enhancing such engagement on Chinese social media.

### Conceptual Model and Research Hypotheses

The elaboration likelihood model (ELM) of persuasion is a theory of information processing that suggests there are 2 routes, central and peripheral routes, to persuasion [22]. In line with the ELM, we systematically distinguished between 2 information processing routes for individual engaging decisions (to engage or not): the peripheral route and the central route. ELM indicates individuals tend to show a higher likelihood of elaboration when they have strong motivations and the ability and individual knowledge to thoughtfully consider the underlying value of social media posts. Here, the central route of the ELM can be used to explain the engaging decision of the users. In contrast, when individuals’ motivations and abilities are limited, the likelihood of elaboration is low and individuals will, in turn, rely on simple cues and second-hand information. Therefore, the peripheral route of the ELM can elucidate users’ engaging decisions.

Importantly, past research suggested that post content characteristics generally influenced motivation and the ability to engage with posts that lead to the central route [23,24]. Common content characteristics include content theme and content mood [25-29]. Content theme is defined as the overarching subject, topic, or central idea that is being conveyed or discussed within the textual content of a post. It is now well established from a variety of studies that content theme plays a critical role in increasing health-related post engagement. Holly and colleagues [25] studied 500 diabetes-related Facebook posts to explore the effect of content themes on engagement behavior. They observed posts related to diabetes symptoms information and positive self-identity had higher sharing while posts related to negative facial expressions, social support, and crowdsourcing had higher commenting. Their findings were consistent with another study [30] that focused on cancer-related posts on Sina Weibo, a Chinese social media platform, which found that social support and cancer treatment attracted the highest number of users and received the greatest number of retweets, comments, and likes. Another recent study [26] collected the COVID-19 pandemic-related videos on one Chinese streaming video platform and identified video content theme emerged as the most critical factor influencing engagement. They observed that emotional-focused messages that psychologically coped with the COVID-19 pandemic situation have more attractiveness compared with problem-focused messages that physically specified risks and strategies. Taken together, these studies suggested content theme is one of the important influencing factors of post engagement, and emotional content (such as social supports) tended to have more engagement in health care communication. Hence, we proposed the first hypothesis:

- The content themes have different effects on public engagement of PEoLC posts on the Chinese social media platform.

Another important central factor of post engagement is content mood. Mood is defined as the emotional tone or atmosphere conveyed by the post content. When individuals are exposed to emotional content on social media, they are likely to share their mood and this further promotes their engagement behaviors, such as sharing, liking, and commenting [31,32]. Previous research established that the influence of post mood differs within various contexts. Bhattacharya et al [27] focusing on Facebook posts from US health agencies accounts found that posts which expressed positive sentiment generated more engagement. However, another study [28] which also focused on US health agencies’ accounts on Twitter found that post mood was not significantly associated with the level of engagement. Xu and Zhang [29] demonstrated that tweets with positive emotions increased the level of retweets, but content showing anger reduced those retweets. Thus, the effect of mood factors differed between diverse platforms, regions, and health topics. This led to our second hypothesis:

- The mood positively affects public engagement of PEoLC posts on the Chinese social media platform.

Furthermore, post mood was possible to moderate the effects of other influencing factors on post engagement. Chen et al [33] analyzed the content published by the Chinese government’s official TikTok account during the COVID-19 pandemic. They demonstrated that the relationship between video content theme and citizen engagement was moderated by the emotional valence of video titles. For instance, videos related to the government handling of the pandemic and guidance for stakeholders that positive titles received a greater number of shares. Likewise, Tang et al [34] supported this view that for a certain type of social media content, positive sentiment can achieve a higher level of engagement behavior. Another study on Sina Weibo [35] found that news related to the COVID-19 pandemic crisis evoking negative emotions had higher engagement than those with positive emotions. Accordingly, the third hypothesis was proposed to test the moderating effect of mood on content theme and post engagement:

- Post mood has a moderating effect on the relationship between content theme and public engagement of PEoLC posts on the Chinese social media platform.

Previous literature [36-40] suggested information richness and source credibility commonly influence post engagement through the peripheral route. Information richness is defined as visual or textual formatting elements (such as visual cues, and external links) that contribute to the comprehensiveness and vividness of information within a post [41]. Many recent studies have shown that information richness may positively affect post engagement. Bhattacharya et al [27] analyzed >45,000 Facebook posts from health agency accounts. They identified Facebook posts with more visual cues, such as photos generated more engagement. This is supported by others who observed significant and robust positive effects of visual cues on post engagement [36,37]. During the COVID-19 pandemic, Ngai et al [38] examined 608 the COVID-19 pandemic posts published by an official news account and found links to external sources fostered sharing behaviors. This led to our fourth hypothesis:

- The information richness positively affects public engagement of PEoLC posts on the Chinese social media platform.

Source credibility is defined as the attractiveness, trustworthiness, and expertise of a post’s publisher according to the source credibility model [42-44]. In the online environment, source credibility was evaluated on the basis of the social cues provided by the publisher in the site network, such as publisher type. Existing literature indicated that user engagement can be affected by different kinds of publishers (eg, health agencies, influencers, and noninfluencers) [39,40,45]. For instance, Kostygina et al [45] tried to investigate source features of Twitter messages in an antitobacco campaign and found using social influencers as message sources could generate more sharable content (eg, campaign hashtags) and greater volume of tweets and reach per day. Xu et al [40] demonstrated that during the COVID-19 pandemic, original posts from official health care accounts on Sina Weibo were more likely to generate more user engagement compared with reposts. Therefore, we proposed the fifth hypothesis:

- The source credibility feature, such as the publisher types have different effects on the public engagement of PEoLC posts on the Chinese social media platform.

**Figure 1:**
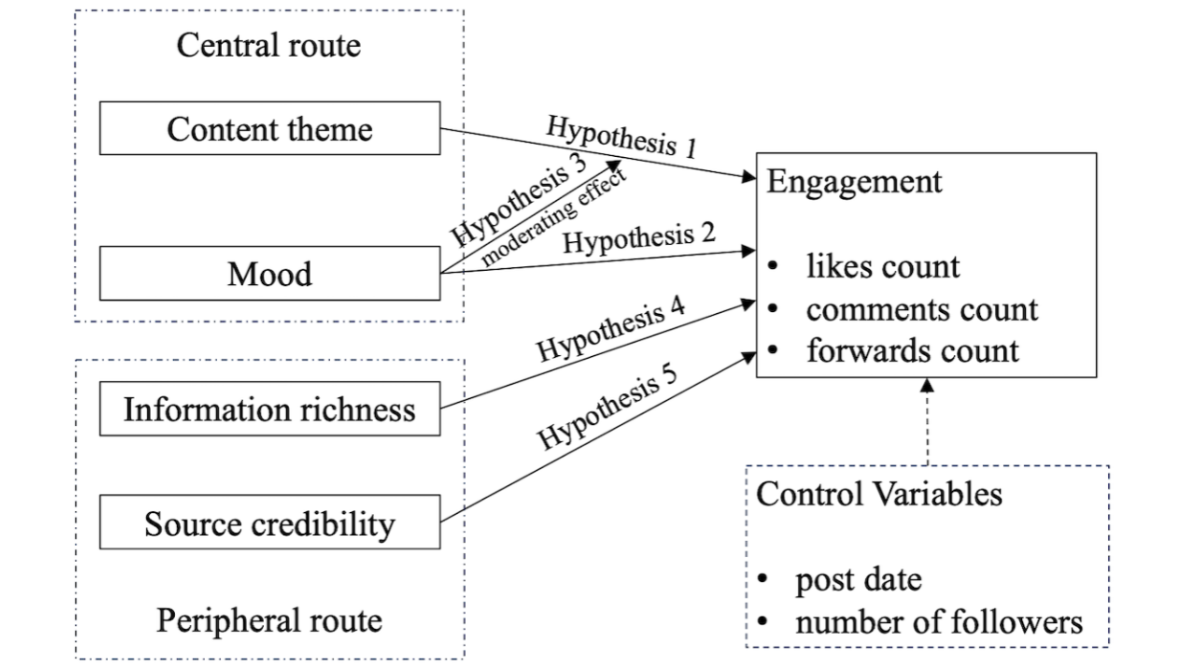
A conceptual framework to explore factors influencing post engagement.

We attempted to understand these influencing factors from the peripheral and the central route. Two dimensions (content theme and mood) of the central route and 2 dimensions (information richness and source credibility) of the peripheral route were selected and tested as shown in Figure 1. Furthermore, according to ELM theory, individuals’ decisions are influenced by their motivation and ability, which determine the different processing routes undertaken. Therefore, in turn, we can also gain some basic ideas of social media users’ motivations and ability for PEoLC content from their preference to the peripheral or the central route when engaging with PEoLC discussion.

## Methods

### Data Collection and Preprocessing

**Figure 2:**
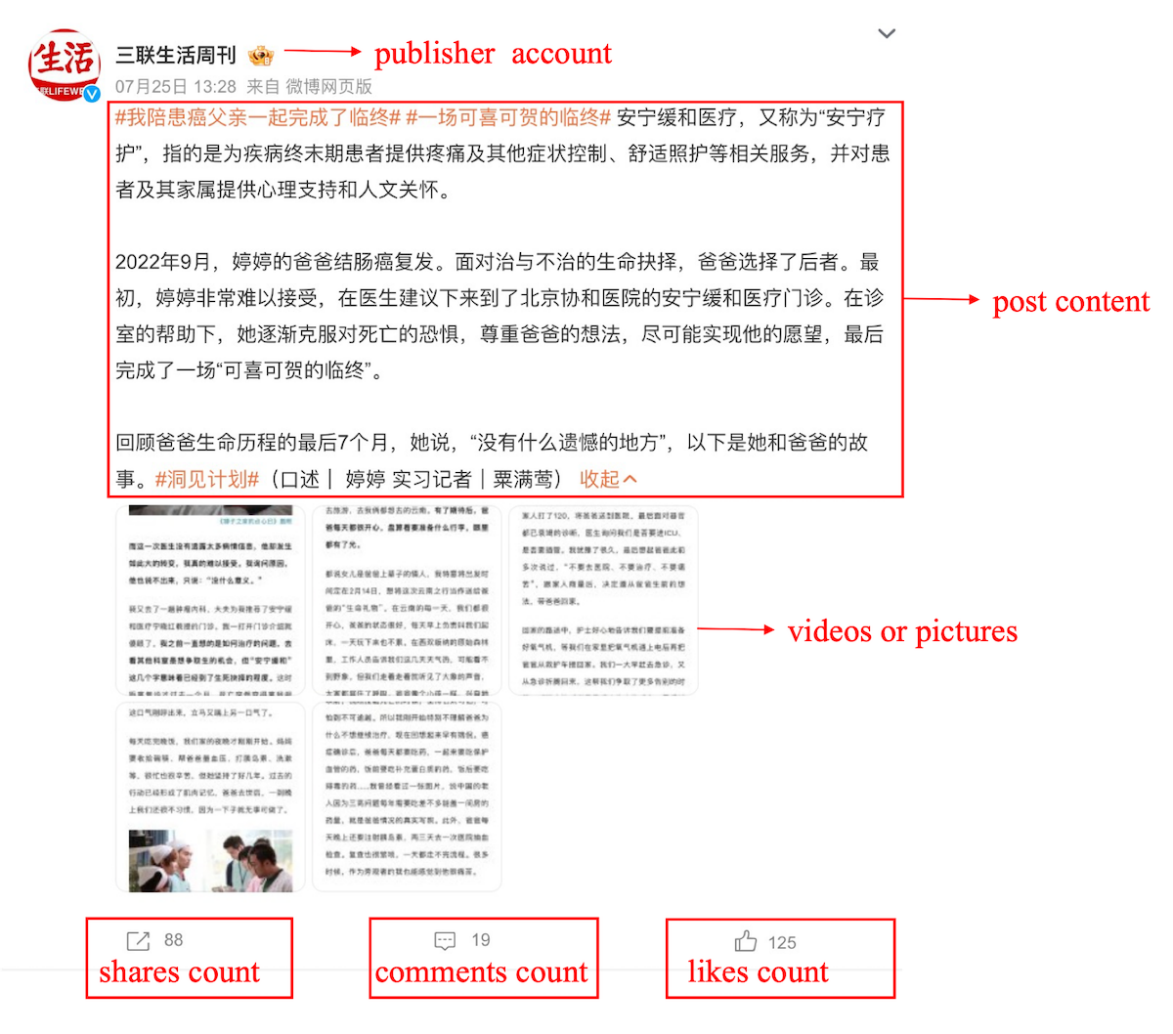
An example of Weibo posts.

We collected Weibo posts dating from September 1 2009 to December 12 2022 through Python (version 3.8.3; Python Software Foundation). Sina Weibo (hereafter referred to as Weibo; Weibo Corporation) is a major social media platform in China, with >575 million monthly active users as of 2024 [46]. It plays a key role in shaping digital discourse and influencing cultural trends in the country. Often described as China’s equivalent of Twitter, Weibo allows users to create short-form content and engage in interactive discussions. User interactions on Weibo primarily include 3 key actions: sharing, commenting, and liking posts. Figure 2 illustrates the typical structure of a Weibo post, providing an example of the platform’s interface and its user interaction mechanisms.

Keywords that were used to retrieve palliative care–related posts, such as “姑息治疗 (palliative care),” “临终关怀 (hospice care),” “生命终末期 (end-of-life)” are listed in Multimedia Appendix 1. To ensure the comprehensive inclusion of palliative care–related keywords, we (1) summarized common keywords used in previous studies [47-49]; (2) consulted experts in palliative care; and (3) conducted a “snowball” search strategy (starting with a small set of search keywords and iteratively expanding it by examining posts retrieved by these keywords and identifying additional keywords). Since the original concepts were in English, YW translated these concepts into simplified Chinese and engaged 2 bilingual Chinese speakers (HZ and YZ) to check translations. Disagreements in wording and meaning were resolved through further discussion.

Data preprocessing was further conducted. First, we detected and deleted posts published by bot accounts. A bot account on a social media platform entails an automated digital entity capable of emulating human interactions, and its potential harms encompass the dissemination of misinformation, manipulation of public discourse, and distortion of authentic engagement dynamics within the digital sociocultural landscape [50]. We identified one account as a bot account if it is abnormally active (published >30 posts a day) [51]. Second, we coped with missing data and outliers. We deleted posts with missing values or outliers in any variables in metadata. There were 2 reasons for doing so. The first was filling in these missing values with a replaced value will inevitably lead to bias. The second was that the total amount of records was large enough to tolerate a very small part of records being deleted [52,53]. Third, we manually went through the rest of the posts and excluded 1565 posts which were irrelevant to PEoLC. Some posts made jokes using PEoLC-related terms, for example: “My phone doesn’t work anymore. I need hospice care for my phone.” Since they cannot contribute to our understanding of PEoLC online discussion, we decided to consider them as noisy data and deleted these posts. Subsequently, we obtained 30,811 palliative care–related Chinese posts published by 20,764 distinct users. All Python (Python Software Foundation) and R scripts (R Foundation for Statistical Computing) used for data collection, preprocessing, and analysis were available from a GitHub (GitHub Inc) repository [54].

### Operationalization of Variables

Public engagement with PEoLC discussion on Weibo was the dependent or response variable of our study and it was reflected in 3 dimensions: sharing, liking, and commenting behaviors. Public engagement could be used to measure the popularity and attractiveness of social media posts [55]. If a post had high engagement on social media, then we call this post *viral* and it has higher exposure. We captured this objective data using web crawlers. An example of how the objective data were shown in a Weibo post is illustrated in Figure 2. In our study, public engagement in a Weibo post (engagement) was calculated by summing the values of these 3 indicators, a common practice in other social media research [55], as follows:

Engagement_i_ = likes count_i_ + shares count_i_ + comments count_i_

Content theme was assessed based on 7 thematic categories, encompassing: personal experiences or stories about PEoLC, educating, discussion about entertainment work (film, television series, and books), reflections on palliative care and death, PEoLC activities or achievements, controversial social news, and fundraising. YW and YZ applied thematic analysis [56] to identify themes. This followed a multistage process, commencing with data familiarization, wherein researchers immersed themselves in the dataset to gain a deep understanding of the content. Subsequently, we generated initial codes, followed by theme development, constant comparison, and refinement of themes to ensure their coherence and relevance. We specifically applied thematic analysis to the content pertinent to PEoLC, ignoring the potential presence of irrelevant content within a post. In cases where a post encompassed multiple themes, the predominant theme was determined on the basis of the theme exhibiting the most distinct characteristics. To ensure analytical rigor, the level of agreement between the 2 reviewers (YW and YZ) for the thematic analysis was determined using Cohen κ. Intercoder reliability by κ coefficient was 0.87 (95% CI 0.89-0.85; *P*<.001). A κ statistic of 0.81 to 1.00 indicates an “almost perfect” strength of agreement. The findings, emerging themes, and their interpretations are presented in Table 1, which provided an in-depth exploration of PEoLC-related topics discussed on Chinese social media.

**Table 1:** Content themes of palliative and end-of-life care (PEoLC) related Weibo posts.

Themes			Example quotes
**Theme 1: personal experiences or stories**			
	Talking about individuals’ experiences with palliative care	“I went to Puren Hospice Hospital for a consultation. The doctor said that my dad can be admitted to their hospital, and if the ward is available my mom can also live in, so that the patient can enjoy the last time with their families. I felt that I have finally found a direction to solve the problem I have been suffering from for a long time.”	
**Theme 2: educating**			
	Elucidating the scientific aspects of palliative care, including its definition, meaning, and development status	“Hospice care is to provide physical, psychological and spiritual care and humanistic services to help patients spend the last journey of their life with dignity by controlling their pain and discomforting symptoms and guiding their family members to get rid of their sadness as soon as possible before the end of their life.”	
**Theme 3: discussion about entertainment work (film, television series, and books)**			
	Discussing palliative care–related films, television series, or books	“It’s quite good to have this type of TV drama. I was even more shocked by the story in episode 7, which was really good. It is about death, the deceased and their families, the funeral home staff, the hospice...We can discuss a lot and feel strength and warmth from the sadness.”	
**Theme 4: reflections on palliative care and death**			
	Reflecting on death or palliative care–related topics	“I am speechless. China is lack of death education and Chinese avoid to face up to life and death, which leads to the next generation who do not understand the value of life and then do not know how to care for the dying. For the sake of ‘filial piety,’ human suffering is prolonged. Repressing human nature is the most antihuman.”	
**Theme 5: activities or achievements**			
	Reports about palliative care activities or achievements	“From 11th to 17th October, the Hospice of the Affiliated Hospital of Guangdong Medical University held a World Palliative Care Day publicity campaign with the theme of ‘Love each other, spread palliative care to help what we need.’ The activities included community and hospital visits and stalls, with the aim of promoting the importance of hospice care, paying attention to the quality of life of poor advanced cancer patients and their families.”	
**Theme 6: controversial social news**			
	A controversial social phenomenon related to palliative care (eg, the legalization of euthanasia in certain countries or protest against the establishment of a hospice center)	“Recently, a community in Xi’an met with collective opposition from owners when it built a care center for the disabled elderly. Many residents said that the hospice affected the quality of life of the residents and hoped that the property owners would choose a new site.”	
**Theme 7: fundraising**			
	Reports on fundraising initiatives and solicits contributions	“The monthly donation programme is now live! Become a monthly donor for palliative care, have one less cup of coffee a month, and share your love and warmth to the children who are sick and undergoing chemotherapy.”	

Mood was measured by a post’s sentiment score. The sentiment score, ranging from 0 to 1, conveyed the negative sentiment to the positive sentiment of a post. We obtained the content of all posts through web crawlers and then applied sentiment analysis to calculate the sentiment scores through Baidu application programming interface (API). The Baidu API, based on deep neural network, was selected since it has better performance in judging the sentiment of palliative care–related posts compared with other popular Chinese sentiment analysis models such as SnowNLP. SnowNLP intrinsically recognized terms such as “death,” and “dying” as negative terms, while these terms were comparatively neutral in the Baidu API. For instance, a post saying “对于剩余寿命无多的终末期患者，姑息治疗主要为患者提供临终关怀及善终服务” (“For patients who are terminally ill with little time left to live, palliative care focuses on providing end-of-life care and hospice services”) obtained a sentiment score of 0.92 through Baidu API while obtaining a sentiment score of 0.07 through SnowNLP.

Information richness was measured by 4 indicators: whether a post had pictures or videos or not, whether a post used hashtags or not, whether a post tagged other users or not and the number of Chinese characters included in a post [38,57]. We obtained the content of all posts through web crawlers first. Then, we applied regular expression in Python to detect pictures, videos, webpage links, hashtags, and tagging other users and applied the count function in Python to calculate the number of Chinese characters.

Source credibility was measured by a post’s publisher type. In empirical social media research, publisher type is frequently used as a proxy for source credibility, as it is readily available and easy to operate [58-60]. While this measure provides a general indication of perceived credibility, it does not encompass the full spectrum of attributes, such as trustworthiness, expertise, and attractiveness. The highly subjective nature of these attributes, coupled with the variability of social media platforms, complicates efforts to capture them through standardized metadata alone. Indeed, previous research has identified a lack of consensus on how to operationalize these measures across platforms [61]. Hence, this study used publisher type to measure source credibility. There were 6 types of publishers: individual users (15,356/30,811, 49.84%), influencers (5312/30,811, 17.24%), governments and hospitals (3265/30,811, 10.6%), enterprises (3133/30,811, 10.17%), media and press (2196/30,811, 7.13%), nonprofit organizations (1549/30,811, 5.03%). We captured the publisher type shown on the publisher’s profile using web crawlers. The difference between individual users and influencers is the social media impact. Influencers are individuals who have a significant following on social media and can influence the opinions and behaviors of their followers. Therefore, they have a greater social media impact compared with other individual users.

In addition, the number of days a post was published and the number of followers that the publisher had were extracted as controlled variables to account for their potential influence on the dependent variable based on previous literature [62,63]. Descriptive statistics of the study variables are summarized in Table 2.

**Table 2:** Descriptive statistics of study variables.

Construct and description				Values
Total number of posts, n (%)				30,811 (100)
**Content theme variables**				
	**Content theme, n (%)**			
		Theme 1: personal experiences or stories	6400 (20.77)	
		Theme 2: educating	4546 (14.75)	
		Theme 3: discussion about entertainment work (films, television series, and books)	4588 (14.89)	
		Theme 4: reflections on palliative care and death	5352 (17.37)	
		Theme 5: activities or achievements	7741 (25.12)	
		Theme 6: controversial social news	767 (2.49)	
		Theme 7: fundraising	1417 (4.6)	
**Mood variable**				
	Sentiment score (ranges from 0, negative, to 1, positive), mean (SD)		0.74 (36)	
**Information richness variables**				
	**Post used hashtags or not, n (%)**			
		Yes	10,275 (33.35)	
		No	20,536 (66.65)	
	**Post tagged other users or not, n (%)**			
		Yes	4355 (14.13)	
		No	26,456 (85.86)	
	Video and image count (number of videos and images included in a post), mean (SD)		1.27 (2.08)	
	Character count (number of Chinese characters included in a post), mean (SD)		248.3 (366.43)	
**Source credibility variables**				
	**Publisher type, n (%)**			
		Individual users	15,356 (49.84)	
		Influencers	5312 (17.24)	
		Governments and hospitals	3265 (10.6)	
		Enterprises	3133 (10.17)	
		Media and press	2196 (7.13)	
		Nonprofit organizations	1549 (5.03)	
**Controlled variables**				
	Days (number of days a post was published), mean (SD)		1283 (998.04)	
	Follower count (number of followers the publisher had), mean (SD)		7.63 (3.45)	
**Dependent variable or response quantity**				
	Comment count (number of comments a post received), mean (SD)		6.182 (123.18)	
	Like count (number of likes a post received), mean (SD)		18.51 (565.39)	
	Forward count (number of forwards a post received), mean (SD)		15.29 (449.35)	
	Engagement (number of comments, likes, and forwards a post received), mean (SD)		39.98 (844.61)	

### Statistical Analysis

Our dependent variable was engagement that exhibited overdispersion (engagement: mean 39.98, SD 844.61; skewness=62.09, kurtosis=5150.66). A significant number of Weibo posts rarely received shares, likes, or comments, while others received a high number in comparison. To deal with this overdispersed count data, we modeled the engagement using negative binomial regression. We first estimated the impact of information richness, source credibility, mood, and content theme on public engagement. The regression model was as follows:

ln(engagement_i_) = Intercept + b_1_content theme_i_ + b_2_mood_i_ + b_3_information richness_i_ + b_4_source credibility_i_

We then explored whether the impacts were contingent upon the emotional valence of each post’s content theme. The regression model was as follows:

ln(engagement_i_) = Intercept + b_1_content theme_i_ + b_2_mood_i_ + b_3_mood_i_ × content theme_i_ + b_4_information richness_i_ + b_5_source credibility_i_

All analyses were conducted using R (version 4.0.2; R Foundation for Statistical Computing).

### Ethical Considerations

As this study used publicly available data, it was exempt from institutional ethics review. To protect participant confidentiality, all personally identifiable information in original posts was anonymized before analysis. Data retention adhered to the principle of minimal necessary storage. In addition, Chinese posts included in the manuscript were translated into English and paraphrased so that they cannot be traced back to the original users.

## Results

### Descriptive Analysis

A total of 30,811 Weibo posts published from September 1, 2009, to December 12, 2022, were used for building regression models. The distribution of these posts’ engagements was largely dispersed, with 14,344 (46.55%) posts receiving 0 engagements (ie, 0 likes, 0 comments, and 0 shares), 15,516 (50.36%) posts receiving 0 to 100 engagements and 951 (3.09%) posts receiving >100 engagements. The annual average engagements of PEoLC-related Weibo posts increased every year from 10.09 in 2012 to 65.57 in 2022. The average engagement of posts varied between post content themes and publisher types. Posts related to theme 6 (controversial social news) had the highest average engagement (mean 205.82, SD 3539.17), followed by posts related to theme 1 (personal experiences or stories; mean 51.28, SD 837.21). Posts related to theme 2 (educating) received the least average engagements (mean 12.18, SD 254.08). Posts published by the media and press attracted the most average engagements (mean 116.17, SD 2326.55), followed by influencers (mean 112.69, SD 1224.51). Posts published by the government and hospital accounts received the least average engagements (mean 7.40, SD 45.19).

### Hypothesis Testing

Table 3 presents the estimates of 2 negative binomial regression models that predicted the engagement of PEoLC-related posts on the Chinese microblogging platform—Weibo. Hypothesis 1 assumed the content themes could have different effects on post engagement. As the content theme was a categorical variable, we treated personal experiences or stories as the reference group. Model 1 presented in Table 3 showed that discussion about entertainment work (films, television series, and books; incidence rate ratio [IRR]=1.37; *P*<.001) and controversial social news (IRR=1.64; *P*<.001) were positively correlated with post engagement, compared with personal experiences or stories. Posts related to educating (IRR=0.48; *P*<.001), reflections on palliative care and death (IRR=0.77; *P*<.001), PEoLC activities or achievements (IRR=0.64; *P*<.001), and fundraising (IRR=0.14; *P*<.001) were negatively associated with post engagement. Consequently, hypothesis 1 was supported.

**Table 3:** Results of the negative binomial regression model 1 and model 2 (with interaction terms).

Variables				Model 1				Model 2		
				IRR^a^		*P* value		IRR		*P* value
Intercept				0.27		<.001		0.24		<.001
**Content theme variables**										
	Theme 1: personal experiences or stories (RF^b^)		—^c^		—		—		—	
	Theme 2: educating		0.48		<.001		0.22		<.001	
	Theme 3: discussion about entertainment work (films, television series, and books)		1.37		<.001		1.49		<.001	
	Theme 4: reflections on palliative care and death		0.77		<.001		0.98		.54	
	Theme 5: activities or achievements		0.64		<.001		0.61		<.001	
	Theme 6: controversial social news		1.64		<.001		2.12		<.001	
	Theme 7: fundraising		0.14		<.001		1.10		.76	
**Mood variable**										
	Sentiment score		0.78		<.001		0.80		<.001	
**Interaction effect**										
	**Content theme** **×** **mood**									
		Theme 1 × sentiment score	—		—		—		—	
		Theme 2 × sentiment score	—		—		2.68		<.001	
		Theme 3 × sentiment score	—		—		0.84		.003	
		Theme 4 × sentiment score	—		—		0.66		<.001	
		Theme 5 × sentiment score	—		—		1.06		.45	
		Theme 6 × sentiment score	—		—		0.36		<.001	
		Theme 7 × sentiment score	—		—		0.11		<.001	
**Information richness variables**										
	Post used hashtags or not		1.39		<.001		1.34		<.001	
	Post tagged other users or not		5.54		<.001		5.94		<.001	
	Videos and images count		1.03		<.001		1.03		<.001	
	Characters count		1.00		<.001		1.00		<.001	
**Source credibility variables**										
	Individual users (RF)		—		—		—		—	
	Influencers		0.90		.01^d^		0.88		<.001	
	Governments and hospitals		0.21		<.001		0.17		<.001	
	Enterprises		0.56		<.001		0.54		<.001	
	Media and press		0.41		<.001		0.41		<.001	
	Nonprofit organizations		0.66		<.001		0.63		<.001	
**Controlled variables**										
	Days		1.00		<.001		1.00		<.001	
	Followers count		1.57		<.001		1.61		<.001	

^a^IRR: incidence rate ratio.

^b^RF: reference factor.

^c^Not available.

Hypothesis 2 proposed the more positive the mood of a post, the more engagement it received. However, model 1 presented in Table 3 showed the sentiment score had a significantly negative association with post engagement (IRR=0.78; *P*<.001). The IRR meant a 1-unit increase in sentiment score could result in a 22% decrease in the number of post engagements. Hence, hypothesis 2 could not be supported by our results.

To test hypothesis 3, we built model 2 to enter the interaction effects between content themes and post mood based on model 1. The results in Table 3, model 2, showed that post mood had a slight moderate effect on the relationship between content type and post engagement. Specifically, theme 2 with positive mood was positively associated with post engagement (IRR=2.68; *P*<.001). When we tested hypothesis 1, theme 2 (educating) was negatively associated with post engagement. This indicates post mood could moderate the effect of content theme on post engagement. Interestingly, only theme 2 with a positive mood was related to high engagement, the other topics with a negative mood were related to high engagement. Hence, hypothesis 3 was supported.

Hypothesis 4 proposed that information richness was positively associated with the level of public engagement of PEoLC posts, namely the post that had more information was likely to receive greater public engagement. Model 1 in Table 3 showed 4 variables representing information richness where all had positive and statistically significant associations with post engagement (used hashtags or not: IRR=1.39; *P*<.001; tagged other users or not: IRR=5.54; *P*<.001; videos and images count: IRR=1.03; *P*<.001; and characters count: IRR=1.00; *P*<.001). The IRR values indicated that posts with hashtags were associated with a 1.09-unit increase in public engagement, while posts tagging other users were linked to a 5.54-unit increase, both compared to posts without these respective features. Hypothesis 4 was therefore supported by the results.

Hypothesis 5 posited the level of public engagement of a PEoLC post was affected by the source, namely the type of publisher. Since publisher type was a categorical variable, we treated individual users as the reference group. Model 1 in Table 3 showed that when compared with individual users, the other publisher types were negatively and significantly associated with post engagement. The IRR values suggested that when compared to posts published by individual users, those from influencers (*P*=.01), governments and hospitals (*P*<.001), enterprises (*P*<.001), media and press (*P*<.001), and nonprofit organizations (*P*<.001) resulted in different levels of decrease in the number of engagements. Consequently, hypothesis 5 was supported.

## Discussion

### Principal Findings

This study represented the first attempt to identify how online discussions promote public engagement in PEoLC in China. Given the prevailing limited awareness of palliative care in low-income regions [64,65], the use of social media as a digital platform for promoting and educating the public about PEoLC is imperative. This study examined the multifaceted factors influencing public engagement with PEoLC-related posts from 4 dimensions (ie, content theme, mood, information richness and source), drawing upon the ELM to expound on these influences. Our results identified that these 4 dimensions exerted significant and distinct effects on engagement. These findings provided a valuable understanding of the existing levels of public engagement with PEoLC-related content on social media. Moreover, they offered guidance to content developers, including event organizers, nonprofit organizations, and academics, on leveraging social media to disseminate research, launch health campaigns, raise funds, and educate about PEoLC to the wider public.

First, our results identified that posts published by government entities, health care institutions, and organizations tend to attract comparatively lower levels of engagement when contrasted with posts from individual accounts. This highlights the potential underuse of organizational accounts in their role of public education and advocacy for PEoLC. Most organizational accounts still adhere to the conventional model of unidirectional communication on Weibo, characterized by didactic scientific presentations and formalized achievements reporting. Effective management and content curation of these accounts are paramount, as they hold the potential to both raise awareness about palliative care and then inspire charitable contributions in support of PEoLC [66].

Furthermore, our analysis indicated that the content theme significantly impacts the level of public engagement with posts. Specifically, we observed that engagement tended to be higher for themes related to entertainment and social matters, while it exhibited a slightly lower level of engagement for informational content. This finding aligns with previous research which demonstrated that content about entertainment on social media tends to elicit greater public engagement [67,68]. This pattern is not solely a manifestation of the intrinsic entertainment and sociable characteristics of social media platforms. It may also be attributed to the fact that leveraging entertainment works (films, books, or television) or current social news as a channel to contact participants offers an efficacious approach for educating individuals about sensitive health topics, such as palliative care within a real-world context [69-72]. By exploring the portrayal of palliative care in books, television dramas, or social news, the public has a chance to be exposed to the concepts of “hospice care” and “palliative care” among others, thereby fostering collective reflection and discussion. For instance, the Chinese television series “Song of Life,” which depicted a palliative professional’s working experiences prompted a discourse concerning the feasibility of establishing hospices in a residential area on Chinese social media. Recent years have witnessed China’s production of some high-quality film and television content about palliative care. Notably, our study reveals that the use of film and television productions to initiate public discourse and engagement on social media platforms has proven to be effective. Given the relatively low acceptance and awareness levels surrounding the topic of palliative care in low-income regions, entertainment education on social media may indeed offer a promising strategy for raising awareness and public understanding and decreasing inequality. The Lancet Commission on the Value of Death also highlighted the significance of the normalization of death communication through mass media and entertainment work [73]. Our results provide empirical evidence for this proposal.

We also identified that the positive mood of a post negatively impacted public engagement around PEoLC-related posts. This is in contrast with previous studies in which a more positive mood in health-related posts attracts more engagement [27]. This phenomenon could be attributed to the sensitive nature of PEoLC, which is related to death and dying. In China, with a general lack of death education and the dominance of traditional Chinese ethical culture, topics related to palliative care are still seen as taboo. The Chinese tradition of filial piety, where children are expected to seek permission from their parents, exerts substantial societal, moral, and public opinion pressure on parental end-of-life decisions [12]. Consequently, it is still difficult for the public to view this concept positively. Therefore, a relatively negative or serious approach to the discussion of PEoLC is more acceptable to the public in the context of Chinese culture, even on social media. Beyond the Chinese cultural context, PEoLC-related posts may display distinctive attributes in comparison to more generalized health care content, warranting further investigation in this area.

Ultimately, our investigation revealed that information richness exerted a positive influence on engagement. Specifically, posts characterized by a greater volume of content, a higher frequency of tagging other users, the incorporation of more hashtags, and the inclusion of more images or videos were correlated with higher engagement levels. This discovery aligns with the outcomes of previous research [27,74-76] in health care communication. In doing so, our study further substantiates a previous observation within the domain of health care social media research, which underscores the notion that the more informative, diverse, and vivid the mode of expression, the greater the attention and engagement garnered by health-related communication in the realm of social media.

According to ELM theory, although post content and mood as central cues could change or form attitude with higher persistence, peripheral cues may play more crucial roles in the context of PEoLC promotion. The ELM theory emphasizes that if the public’s motivation or ability to process the issue-relevant information is low, persuasion can occur by a peripheral route in which processes invoked by simple cues in the persuasion context influence attitudes [77]. Considering the relatively limited awareness and understanding of PEoLC among the Chinese public, stakeholders may benefit from using peripheral routes, such as information richness and source credibility when promoting PEoLC on online platforms.

Recent research suggests that older adults in China prefer accessing palliative care information through electronic media [78]. This finding underscores the potential of online platforms, including social media, as effective tools for promoting and educating the public about PEoLC. While older adults may not be frequent users of social media, young social media users, often their significant others, can play a critical role in disseminating PEoLC information, influencing health care decisions at both individual and community levels. This, in turn, can enhance the overall quality of end-of-life care within society. In the Chinese cultural context, where end-of-life care decisions often involve multiple stakeholders, including family members and health care providers, engaging diverse groups through social media can help facilitate the integration of PEoLC into the broader health care system. Social media platforms, therefore, hold significant potential for enhancing public understanding and improving the quality of care for vulnerable populations, such as the elderly.

### Implications and Strategic Suggestions

On the basis of the study findings, we developed some practical strategies to help individuals or organizations to boost public engagement about PEoLC-related discussions or campaigns on social media platforms (Textbox 1 [79]). These strategies suggest content developers or account operators follow certain patterns during the content development process. These recommendations provide valuable guidance, particularly for organizational accounts, which may face challenges in attracting engagement. This bears special significance for nonprofit organizations, where social media accounts may serve as a pivotal channel for fundraising. As our findings were based on Chinese social media platforms, these strategies may work more effectively within similar cultural contexts and PEoLC development levels.

**Practical strategies to help individuals or organizations to boost public engagement about palliative and end-of-life care (PEoLC)-related discussions or campaigns on social media platforms.**

- Use trending entertainment works and social news as a trigger—when developing PEoLC-related content on social media, the strategic incorporation of trending entertainment works and social news may be a compelling approach to enhance user engagement. Content developers can use entertainment works or social news as a lead-in to start their content or only attach these trending hashtags (such as #Song of Life##Do you agree to have a hospice in your community#) in their posts. The inherently captivating nature of trending topics in entertainment and social events provides a dynamic backdrop against which PEoLC-related content can be situated, sparking user curiosity and interaction. By using trending entertainment works and social news as catalysts, content creators can intrigue the audiences thinking in a real situation and thus achieve their intended purpose of promoting or educating.
- Try to engage influencers in promotion or education—influencers, often recognized for their expansive reach and persuasive capacities, offer a unique opportunity to enhance the visibility and impact of PEoLC messaging on social media. Concrete strategies to realize this encompasses collaborating with influencers who have a personal connection to PEoLC, encouraging them to share personal narratives, insights, and experiences, and endorsing PEoLC resources, materials, and campaigns on their channels. Organizations must realize that it is not enough to limit content distribution merely to their followers, who have already shown interest in the topic of PEoLC. Rather, organizations could interact with social media influencers who have much more diverse followers, to engage a wider audience with little knowledge of PEoLC. In addition, organizations could hold influencer-led awareness campaigns, live question and answer sessions, and educational webinars on both organizations’ and influencers’ accounts. By harnessing the influence of these advocates, organizations are more likely to significantly amplify their reach and raise public awareness of PEoLC on social media.
- Content works better with visual cues—visual elements, such as figures and videos, not only enhance the esthetic appeal of posts but also play a pivotal role in conveying complex concepts and eliciting emotive responses. Visual cues provide audiences with the opportunity to firsthand experience the dilemmas faced by stakeholders in PEoLC and immerse themselves in the personal narratives of these individuals, thus fostering an understanding of the profound significance and relevance of PEoLC. These digital affordances (eg, geotags, images, videos) provide vulnerable groups such as PEoLC stakeholders with an opportunity to gain more exposure on social media and then raise public awareness. Moreover, visual elements can transcend language barriers and resonate on a universal level, further broadening the reach and resonance of the PEoLC message globally. In this context, it is necessary for palliative care communication to incorporate visual elements to enhance understanding, foster empathy, and increase engagement on social media platforms.

### Limitations

While the results of this research provide valuable insights, they must be viewed in light of certain limitations. The primary limitation pertains to the use of Weibo as the sole platform for analyzing PEoLC-related posts. While Weibo is one of the most extensively used social media platforms in China, with a user base exceeding 575 million monthly active users, it represents just one facet of the broader social media landscape. However, the platform’s significant reach and influence in the Chinese context provide a robust foundation for the study, thereby mitigating the potential limitation of not, including other platforms. In addition, the process of keyword selection for retrieving relevant posts on Weibo presented its challenges. Despite our rigorous approach, using 3 diverse methods to ensure a comprehensive inclusion of keywords, there remains a marginal possibility that certain terms used in informal contexts might have been overlooked. It is also noteworthy that terms such as “euthanasia” were deliberately excluded from our search parameters. Although sometimes associated with hospice care in China, the term is often misinterpreted as “suicide” in social media discourse. This strategic exclusion was aimed at reducing the influx of irrelevant data, thereby enhancing the relevance and quality of our dataset. A further limitation is that the operationalization of source credibility based solely on publisher type may not fully capture the perceived trustworthiness, expertise, or attractiveness of the source. While publisher type is commonly used in previous studies, it provides only a partial view of credibility in social media environments. Future research should explore additional or alternative variables to more comprehensively assess source credibility. Besides, the extensive data collection period (2009-2022) introduces potential variability in the results due to the evolving social media landscape and changing user behaviors over time. However, existing literature suggests public attitudes and behaviors toward PEoLC remained relatively stable during this period [3,80]. While aggregating engagement factors over the years may potentially obscure period-specific patterns, it provides a comprehensive overview of long-term trends. Moreover, this extended timeframe ensures a substantial dataset, which improves the statistical power, generalizability, and robustness of the regression models [81]. Nevertheless, future studies may benefit from temporal segmentation to capture nuanced shifts in engagement patterns across different periods. We also consider a multimodel research by incorporating text, image, and video content analysis in detecting engagement patterns in the future.

### Conclusions

While death is a universal and inevitable experience, discussions surrounding it are often avoided in China. This reluctance highlights the urgent necessity to enhance public understanding and awareness about PEoLC in the country. This study pinpoints social media as a promising tool for advancing PEoLC and igniting public engagement on related topics within the Chinese context. Drawing from the ELM theory, our study unveils distinct characteristics of PEoLC-related posts on social media that capture public attention. Acknowledging these unique traits, we have formulated targeted strategies aimed at bolstering public involvement in PEoLC discourse. These strategies include leveraging influencers, using entertainment or social news as catalysts, and integrating visual cues. By adopting these approaches, organizations can effectively communicate with the community, promoting PEoLC in a manner that is both accessible and compelling to the public. This study also holds the potential to catalyze a broader cultural shift toward open and constructive conversations about death and dying in China.

## Appendix

Multimedia Appendix 1 Search keywords used to retrieve related posts.

## Abbreviations

API: application programming interface
ELM: elaboration likelihood model
IRR: incidence rate ratio
PEoLC: palliative and end-of-life care

## Data Availability

Given the potentially sensitive nature of the social media data, which may contain individual-specific information, the dataset generated and analyzed in this study are available from the corresponding author only on reasonable request.
