# Supplementary material for: Promoting Public Engagement in Palliative and End-of-Life Care Discussions on Chinese Social Media: Model Development and Analysis
Source: J Med Internet Res. 2025 Mar 18;27:e59944. doi: 10.2196/59944 (PMC11962336; doi:10.2196/59944)
Supplement: Multimedia Appendix 1 [file jmir_v27i1e59944_app1.docx]

**Table 1. Search keywords used to retrieve related posts.**

| **Concepts in English** | **Search keywords in Chinese** |
| --- | --- |
| palliative care | 姑息治疗，舒缓疗护，舒缓治疗 |
| palliative medicine | 姑息医学，姑息医疗，舒缓医疗，舒缓医学，缓和医疗 |
| hospice care | 临终关怀，临终照护，临终护理，宁养照护，宁养服务，安宁照护，安宁疗护 |
| hospice home | 疼痛病房，安宁病房，宁养院，宁养医院 |
| hospice practitioner | 安宁医生，安宁护士 |
| bereavement support/care/services | 丧亲关怀 |
| grief counseling/support/work | 哀伤辅导，哀伤咨询，哀伤关怀 |
| supportive care | （无） |
| end-of-life care / terminal care | 终末期照护 |
| end of life | 生命终末期 |
| comfort care | （无） |
| spiritual care | 灵性照护，灵性照顾 |
| advance cancer care | 癌症终末期照护，癌症末期照护，癌症晚期照护 |
| ACP (Advance Care Planning) | 预立医疗照护计划 |
| advance directive | 预立医疗指示，预先医疗指示，预设医疗指示 |
| living will | 生前预嘱，医学预嘱，预嘱 |
| death with dignity | 尊严死，死的有尊严，死得有尊严，死地有尊严，有尊严地死，有尊严的死，有尊严得死 |
